# Increased Survivorship and Altered Cytokine Profile from Treatment of Influenza A H1N1-Infected Mice with Ekybion: A Drug Complex of Natural Extracts and Inorganic Compounds

**DOI:** 10.1155/2011/192079

**Published:** 2010-10-19

**Authors:** Christopher Lupfer, Didier Besnouin, Samuel E. Tepper, Maciej Maselko, Kristin M. Patton, Manoj Pastey

**Affiliations:** ^1^Genetics Program, College of Agricultural Science, Oregon State University, Corvallis, OR 97331-7303, USA; ^2^Department of Biomedical Sciences, College of Veterinary Medicine, Oregon State University, Corvallis, OR 97331-4801, USA; ^3^Equine Veterinary Clinics and Laboratory of Dr Besnouin, DVM, 61310 La Cochère, France; ^4^Molecular and Cell Biology Program, College of Science, Oregon State University, Corvallis, OR 97331, USA

## Abstract

Ekybion is a drug complex of 16 natural extracts and inorganic compounds designed to treat a variety of respiratory pathogens of bacterial and viral origin. It is licensed throughout Europe for the treatment of respiratory tract infections from equine parainfluenza type 3 and equine herpes virus type 1 in equine stables. The purpose of this paper was to test the efficacy of Ekybion on a well-developed animal model of influenza A infection and determine a mode of action. Experiments were performed with Balb/c mice infected with a lethal dose of influenza A/PR/8/34 H1N1 virus and treated with nebulized Ekybion every 8 h in a time-dependant or dose-dependant fashion. These experiments showed that mice treated prior to infection with Ekybion had a higher survival rates (~46%) compared with untreated animals (~0%). Paradoxically, these mice showed no significant difference in lung virus titer or weight loss. There was, however, a decrease in the level of GM-CSF, IL-6, and G-CSF cytokines in the lungs of Ekybion-treated, infected mice. It is possible that decreases in proinflammatory cytokines may have contributed to increased survivorship in Ekybion-treated influenza-infected mice.

## 1. Introduction

Influenza A virus is an enveloped, single stranded, negative sense, segmented RNA virus belonging to the *Orthomyxoviridae* family. Influenza A infection is of particular concern to the human and veterinary medical communities. Seasonal outbreaks in humans result in hundreds of thousands of deaths annually; many are due to secondary bacterial infections [1]. In addition, there are frequent outbreaks of equine and canine influenza, especially among racing animals housed in large groups, which facilitates spreading of the infection [2, 3]. Further concerns are raised by the ability of influenza A viruses to undergo genetic reassortments potentially creating new variant virus with zoonotic consequences such as the ongoing transmission of avian influenza strains to humans [4–6] and equine strains to dogs [7]. Most recently, the global pandemic of swine origin influenza A H1N1 has resulted in millions of confirmed infections and ~17,000 thousand confirmed fatal cases [8]. 

Each year, seasonal influenza epidemics kill approximately 36,000 people in the USA and 250,000–500,000 people worldwide [9]. However, this is a complicated disease where not just viral titers but the inflammatory reactions are a major cause of morbidity and mortality [10–12]. The systemic and broche alveolar production of proinflammatory cytokines is a well-established feature of influenza A infections with significantly increased levels of TNF-*α*, IL-1*β*, IL-6, IL-8, IL-10, RANTES, MCP-1, IFN-*α*, and IFN-*γ* in the nasopharyngeal fluids and plasma of patients [13–19]. Most of the strategies explored to date for controlling or treating influenza have focused on controlling the spread of the infection and the use of vaccines or antiviral agents, rather than combining antiviral agents with treating the lung and systemic inflammatory reactions that occur during infection. 

Many natural compounds have been studied for their potential immunomodulatory and antiviral effects [20–22]. Natural compounds have been widely accepted and used for centuries and continue to be used, especially in developing countries, where access to allopathic medicine is limited due to availability and cost. However, clinical and preclinical testing of alternative medicines to scrutinize their purported uses is needed to validated alternative therapies just as it is needed to validate allopathic medicine [23]. The presence of flavonoids, flavones, and other polyphenolic compounds in many natural remedies often endows them with their observed therapeutic benefits [24]. Many natural compounds have been examined for their antiviral or anti-inflammatory effects during influenza A virus infection, and the active components in many cases have been isolated. Some of these include the traditional Chinese medicines *Evodia rutaecarpa* [25] and *Sophora flavescens* [26], as well as several plants commonly used in Nepalese traditional medicine [27]. 

In our paper we have examined the effects of Ekybion, a drug complex developed to treat infections and inflammation of the respiratory tract of horses caused by a wide variety of infectious agents. Ekybion is a mixture of 16 inorganic compounds and plant extracts commonly used in European Pharmacopea [28, 29] and has been used throughout Europe for nearly two decades for the treatment of equines with various viral infections including equine herpes virus type 1 and equine parainfluenza virus type 3 [30]. Previous research performed at the Pasteur Institute of Paris showed that Ekybion is capable of inhibiting the growth of several viruses *in vitro* including equine herpes virus type 1 (rhinopneumonitis), reovirus, rhinovirus, equine arteritis virus (Bucyrus strain), and human parainfluenza virus type 3 (A. Jacquet, Pasteur Institute, personal communication). However, Ekybion has yet to be tested in a controlled laboratory setting against influenza A virus nor has it undergone clinical evaluation for efficacy in human diseases. We show here that when used appropriately, Ekybion reduces mortality in mice infected with influenza A/PR/8/34 H1N1 virus.

## 2. Materials and Methods

### 2.1. Virus and Cells

Madin-Darby canine kidney (MDCK) cells were maintained at 37°C with 5%  CO_2_ in MDCK growth medium consisting of minimum essential media (MEM) (Hyclone, Logan, UT) supplemented with 100 U/mL penicillin, 100 *μ*g/mL streptomycin (Gibco, Invitrogen, Carlsbad, CA), and 10% fetal bovine serum (FBS) (Hyclone). Influenza A/PR/8/34 H1N1 was obtained from ATCC and grown in MDCK cells in virus growth medium consisting of MEM supplemented with 100 U/mL penicillin, 100 *μ*g/mL streptomycin, and 1.0 *μ*g/mL TPCK-treated Trypsin (Sigma-Aldrich, St. Louis, MO). Virus was harvested 2 days postinfection and stored at −80°C for future use. Virus was titered by standard plaque assay on MDCK cells (see Virus titer determination).

### 2.2. Mice

All animal experiments were performed using 6–8-week-old female Balb/c mice, each weighing approximately 15–18 g (Simonsen Laboratories, Gilroy, CA). Mice were allowed food and water ad libitum throughout the studies. All animal experiments were carried out under biosafety level 2 conditions with protocols approved by the Institutional Animal Care and Use Committee of Oregon State University.

### 2.3. Preparation of Ekybion

Ekybion consists of 16 pure inorganic compounds or plant extracts prepared individually in 45–95% ethanol. An example of one extraction procedure is given as follows: whole flowers from Arnica Montana were ground and extracted in 45% ethanol in purified water. Extracts were then examined by thin-layer chromatography on silica plates using chloroform : glacial acetic acid : methanol : purified water in 15 : 8 : 3 : 2 ratios, respectively, as the solvent. Chromatography plates were allowed to run their length, dried, and then examined under UV light at 365 nm. Under these conditions, 4 blue bands and 1 red band are visible at relative distances of 35, 45, 75, 95, and 98%, respectively. Following extraction, the 16 individual solutions are combined together with purified water and sterile filtered. To make the working stock (1X), the concentrated stock complex is diluted 1 : 500 in purified water containing 0.2% ethanol. The final working stock contains the 16 components in purified water with a total of 0.4% ethanol (see Table 1). 

### 2.4. Efficacy of Ekybion in Cell Culture

MDCK cells were grown in 24-well plates until confluent and then infected with 1000 plaque forming units (PFU) of influenza A/PR/8/34 H1N1 virus in 50 *μ*L at 37°C for 1 hour with periodic shaking. Infection medium was removed, and 500 *μ*L of virus growth medium was added (MEM supplemented with 0.5 *μ*g/mL amphotericin B, 100 U/mL penicillin, 100 *μ*g/mL streptomycin, and 1 *μ*g/mL TPCK-treated trypsin). Quadruplicate wells were treated at 1 h preinfection or 1, 2, 4, or 12 hours postinfection by adding 25 *μ*L Ekybion to each well making a 1 : 20 dilution. Medium was collected at 24 and 48 hours postinfection and virus titer determined by hemagglutination assay (see virus titer determination). 

### 2.5. Efficacy of Ekybion In Vivo

Mice were infected with 1000 PFU (approximately 5 mouse lethal dose 50 (5MLD_50_)) of influenza A/PR/8/34 H1N1 in 100 *μ*L PBS by intranasal administration (IN) following anesthesia with 67 mg/Kg Ketamine and 4.5 mg/Kg Xylazine. Ekybion was delivered through inhalation to mice by means of a nebulizer (Inspiration Nebulizer, Respironics New Jersey, Inc., Parsippany, NJ). All mice were treated with Ekybion every 8 hours for ten days by directly holding their nose over a stream of nebulized Ekybion as indicated below. Two to five mice in each group were euthanized by isoflurane followed by cervical dislocation on day two postinfection for removal of lung tissue for virus titration by plaque assay and histological examination. In addition, mice were weighed daily for 16 days to determine illness and time to death or recovery. 

Determination of dose response was carried out by treating mice with different concentrations (1X or 10X) of nebulized Ekybion for the same duration of time, or by treating with the same concentration for different time durations (2 or 4 minutes). All dose response groups began treatment at 3 hours prior to infection and were treated every 8 hours for 10 days. For time response experiments, mice were treated with the same dose of Ekybion (1X) but starting at different time points before or after infection (3 hours preinfection and 6 hours postinfection) and continuing every 8 hours for 10 days.

### 2.6. Toxicity of Ekybion

20 mice were treated with 1X Ekybion 3 times daily for 10 days. Mice were weighed daily and monitored for illness. On day 4 of treatment, 5 mice were euthanized and lung tissue collected for histopathological examination (see Histopathological examination of lung tissue). Additionally, groups of 2 mice each were treated with various concentrations of Ekybion from 1X to 250X, or 0.4% ethanol in diH_2_O (control), by holding noses of mice directly over nebulized Ekybion for 2 minutes, every 8 hours, for three days. All mice were weighed daily and monitored for signs of illness.

### 2.7. Virus Titer Determination

To determine the titer of virus from cell culture experiments hemagglutination assay was performed by making twofold serial dilutions of the collected medium in 50 *μ*L of phosphate buffered saline (PBS) in round bottom 96-well plates. 50 *μ*L of 0.5% chicken red blood cells (PML Microbiologicals, Portland, OR) in PBS was then added to each well and the plates incubated at room temperature for 1 h. Virus titer was determined as the highest dilution in which duplicate wells showed greater than 50% hemagglutination. 

Virus titers in lung tissue homogenates were determined as reported previously [31] by making ten-fold serial dilutions and infecting monolayers of MDCK cells in duplicate wells of 24-well plates. Titers were determined by the highest dilution in which duplicate wells contained at least 5 plaques per well. Plaque counts were then normalized based on lung weights and all lung titers are presented as PFU/g lung.

### 2.8. Histopathological Examination of Lung Tissue

Sections of lung tissue were fixed in 4% neutral buffered paraformaldehyde, embedded in paraffin and sectioned at 5 micron thickness via routine processing. Sections were stained with hematoxylin and eosin. All sections were evaluated by a veterinary pathologist (Kristin M. Patton). Tissue changes were subjectively ranked according to degree of inflammation, extent of inflammation, and degree of necrosis (0 = no inflammation, 1 = mild inflammation, 2 = moderate inflammation, and 3 = severe inflammation).

### 2.9. Cytokine Levels in Lungs

Cytokine levels in mouse lungs were determined using RayBio Mouse Cytokine Antibody Array I (RayBiotech, Norcross, GA**)**, which tests for 22 mouse cytokines. Procedure was performed per manufacturer's instructions using 500 *μ*L mouse lung homogenate (see Virus titer determination) diluted 1 : 2 in blocking buffer. Arrays were imaged and analyzed on a ChemiDoc XLS using Quantity One software (Bio-Rad, Hercules, CA). Additional cytokine analysis was also performed using the mouse cytokine 22-plex kit from Millipore (Billerica, MA) and analyzing on a Luminex 200 platform (Luminex Corporation, Austin, TX) per manufacturer's recommendations.

### 2.10. Statistics

All statistical analyses were performed using GraphPad Prism version 4.00 for Windows (GraphPad Software, San Diego, CA). Two-way ANOVA with Bonferroni posttest was used to determine significance of *in vivo *toxicity by weight loss. One-Way ANOVA with Dunnett's posttest was used to test for treatment effect on virus titer and histopath scores. Student's *t*-test was used for cytokine expression profiling, and log-rank (Mantel-Cox) test and Gehan-Breslow-Wilcoxon test were used for determining the significance of survivorship.

## 3. Results

### 3.1. In Vitro Efficacy

**Figure 1:**
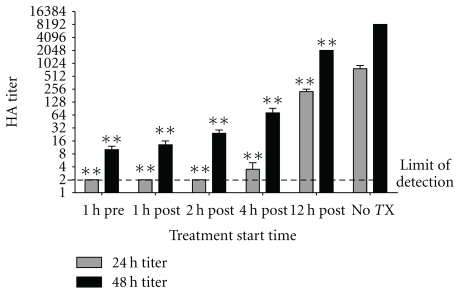
*In vitro* efficacy of Ekybion. Inhibition of influenza A in MDCK cells was performed by treating cells infected with 0.01 MOI influenza A/PR/8/43 H1N1 begnining at the indicated times pre- or postinfection and measuring virus titer by hemagglutination assay at either 24 (grey bars) or 48 (black bars) hours postinfection. (*n* = 4, **P < .001 by Two-Way ANOVA with Bonferroni posttests on Log2 transformed data).

Although Ekybion has been used in Europe to treat respiratory diseases in equines, it has never been empirically tested for its efficacy on influenza A in a controlled laboratory setting. Based on previous research with Ekybion (A. Jacquet, Pasteur Institute, personal communication), we tested the ability of a 1 : 20 dilution of the working stock of Ekybion in virus growth medium to inhibit influenza A/PR/8/34 H1N1 strain in MDCK cells. Treatment began at different times before or after infection (1 h preinfection, or 1, 2, 4, or 12 h postinfection). Control cells were treated with a 1 : 20 dilution of ddH_2_O containing 0.4% ethanol in virus growth medium. Samples were taken at 24 h and 48 h postinfection, and influenza titer was determined by hemagglutination assay. Pretreatment or posttreatment of MDCK cells significantly reduced virus growth for at least 48 h when treatment was initiated by 12 hours postinfection (Figure 1). Inhibition of influenza was most prominent when treatment was performed prior to or within 4 h after infection. These results indicate that Ekybion is capable of significantly reducing influenza A/PR/8/34 H1N1 virus growth in cell culture when treatment is started within the first 12 h of infection.

### 3.2. Toxicity

**Figure 2:**
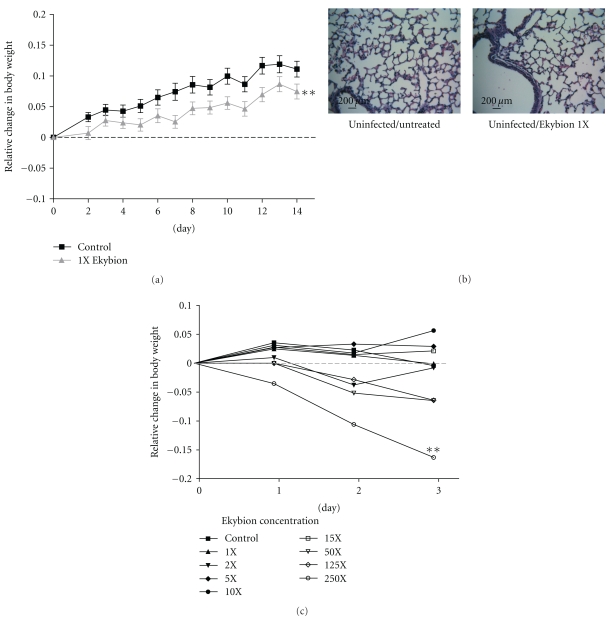
Toxicity of Ekybion in Balb/c mice. (a) Twenty uninfected 8-week old female Balb/c mice were treated with 1X Ekybion every 8 hours for 10 days. Toxicity was determined by changes in daily weight compared to twenty untreated control animals. (b) Two animals from each group were euthanized on day 4 of treatment and lung tissue collected for histological examination for tissue damage or inflammation. Histological examination revealed no inflammation in treated or untreated control animals. (c) Two mice per group were treated every 8 hours for three days with Ekybion at the indicated concentrations and weighed daily to determine toxicity. (**P < .01 by Two-Way ANOVA with Bonferroni posttests).

Initially we tested the toxicity of 1X nebulized Ekybion by treating one group of mice three times daily for 10 days and comparing them to untreated controls. We observed that 1X Ekybion treated mice did gain slightly less weight than control animals (Figure 2(a)) but showed no other effects and had normal lung tissue compared to controls (Figure 2(b)).We next tested various doses of Ekybion ranging from 1X to 250X and treated mice for three days with nebulized Ekybion. No toxicity was observed until a concentration of 50X, and significant toxicity was not observed until 250X, resulting in a 16% decrease in body weight (Figure 2(c)). Together, these results indicate that there was no toxicity associated with Ekybion at the concentration intended for clinical use (1X).

### 3.3. In Vivo Efficacy

**Figure 3:**
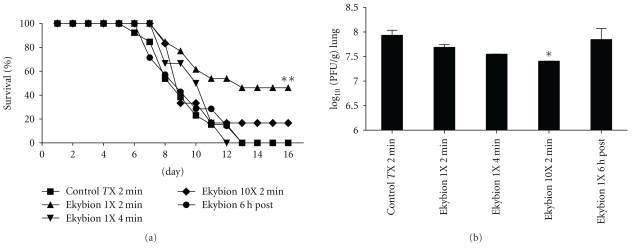
Effects of Ekybion on influenza A infection in Balb/c mice. Female Balb/c mice were infected with 5 MLD_50_ influenza A/PR/8/34 H1N1 and treated every 8 hours for 10 days beginning at indicated times and doses. Mice were weighed daily (data not shown), and monitored for survival for 16 days (a). On day 2 postinfection, 2–5 mice per group were euthanized for determination of virus titer in lungs by plaque assay (b). There were no significant differences in weight loss or lung pathology between any groups. However, there was a dose-dependant antiviral response with significant reduction in virus titer seen in the 10X group (*P* < .05 by one-way ANOVA with Dunnett's posttest). Survival in the 1X 2 min group was also significantly improved (**P < .01 by log-rank (Mantel-Cox) test and Gehan-Breslow-Wilcoxon test).

Next, we evaluated the efficacy of Ekybion at inhibiting influenza virus in infected mice. Doses were administered by either increasing the time of the treatment (2 min versus 4 min) or by keeping the time constant (2 min) and increasing the concentration of the Ekybion used in the nebulizer (1X versus 10X). Groups of mice were infected with 5MLD_50_ influenza A/PR/8/34 H1N1 and treated with various concentrations of Ekybion beginning at 3 h preinfection and continuing every 8 h for ten days in all groups. The control group was treated with 0.4% ethanol in ddH_2_O. Additionally, one group was treated with 1X Ekybion for 2 min each starting at 6 h after infection. Mice were weighed daily for 16 days and 2–5 mice were sacrificed on day 2 postinfection to determine the virus titer in lung tissue. We saw no significant differences in weight loss (data not shown). We also observed that treatment with 1X Ekybion for 2 min (the lowest dose) was the most effective with a 46% survivor rate compared to 0% in the control treatment group (Table 2 and Figure 3(a)). However, treatment with higher doses (10X) or treatment for longer periods of time (4 min) had no beneficial effect on survival. We did observe a dose response effect on virus titer with the 10X 2 min treatment group having a significant decrease in virus titer of 0.53 Log_10_ (Figure 3(b)). In all, our results indicate that the intended 1X dose of Ekybion used in a prophylactic manner may reduce mortality in mice inoculated with a lethal dose of influenza A/PR/8/34 H1N1 but not by reducing virus titers.

### 3.4. Immunomodulation

**Figure 4:**
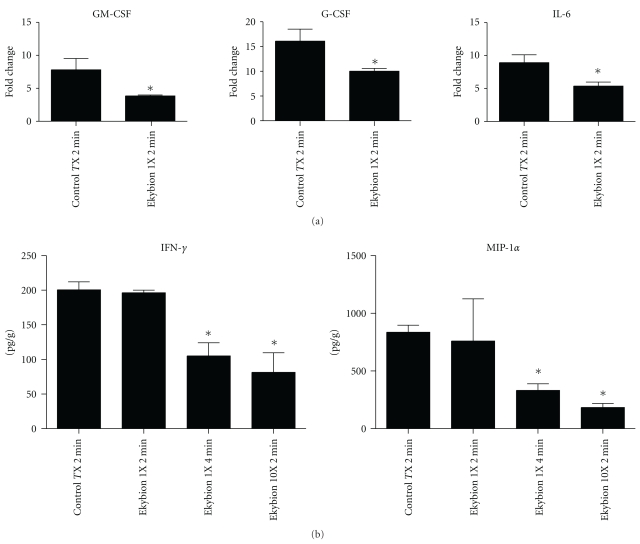
Immunomodulatory effects of Ekybion. Lung tissue previously harvested at day 2 postinfection was homoginized and tested for expression levels of 22 cytokines. Only those cytokines with significant changes are shown. (a) Expression levels were determined initially by membrane-based antibody array (RayBio) and normalized to uninfected untreated control animals. (b) Subsequent analysis was performed by multiplex bead-based cytokine detection (Millipore), and expression levels are displayed as pg/g lung tissue. 2 min = 1X treatment for 2 min, 4 min = 1X treatment for 4 min, and 10X = 10X treatment for 2 min (*P < .05 by Student's *t*-test, *n* = 2-3 per group).

Lung tissue homogenates collected at 2 days postinfection were utilized to determine the cytokine levels at the site of infection. Mice treated with Ekybion displayed a general trend toward lower cytokine production with significant decreases in GCSF (6.0-fold decrease), GM-CSF (4.0-fold decrease), and IL-6 (3.5-fold decrease) in the 1X 2 min treatment group (Figure 4(a)). Further decreases were generally seen in the 1X 4 min group or the 10X 2 min group with additional significant decreases in IFN-*γ* and MIP-1*α* (Figure 4(b)). Based on these lung cytokine levels, it appears that Ekybion may modulate the immune response to influenza A infections *in vivo* in addition to having a direct antiviral effect. It is possible that higher concentrations (10X) of Ekybion may have proved ineffective due to excessive immunosuppression which may have negated any benefit from reduced virus growth.

## 4. Discussion

Ekybion was specifically designed to provide treatment which is effective on a wide variety of infections of different origins by combining a number of plant extracts and inorganic compounds suspected to possess antimicrobial and/or immunomodulatory properties.* Echinacea angustifolia* has been shown to be the most potent *Echinacea* species with respect to immune modulation [32, 33]. Recent studies show that *Echinacea * has both anti-inflammatory and anti-influenza effects [10]. *Arnica montana* has also been shown to possess anti-inflammatory and antimicrobial properties mediated by the presence of two compounds, namely thymol and helenalin [34, 35]. Thymol is capable of inhibiting elastase release from activated neutrophils, possibly reducing some of the collateral damage associated with inflammation [36] and inhibiting influenza A replication due to nonspecific inhibition of protein synthesis [37]. Helenalin's anti-inflammatory properties are mediated through inhibition of NF-*κ*B signaling by alkylating the p65 subunit [38]. Though not specifically demonstrated for helenalin, it has been shown that inhibition of NF-*κ*B results in inhibition of influenza due to a decrease in caspase 3 activity, which is necessary for the nuclear export of viral ribonucleoprotein complexes [39–41]. Finally, *Thuja occidentalis* extracts have been extensively studied alone or in combination with other plants for their antimicrobial and immunomodulatory properties, and a review was recently published in this journal [42]. In all, there are several components present in Ekybion that have known antimicrobial and immunomodulatory properties which would be beneficial for the resolution of inflammation and infections.

We have shown here that Ekybion treatment of mice infected with influenza A/PR/8/34 H1N1 results in an increase in survivorship of 46% over control groups if administered at the intended 1X concentration and in a prophylactic manner. In this model, influenza-infected mice treated with Ekybion showed significant decreases in GCSF, GM-CSF, and IL-6 (Figure 4(a)). Further significant decreases were also seen in the 1X 4 min or the 10X 2 min groups with IFN-*γ* and MIP-1*α* (Figure 4(b)). In addition, we observed a dose dependant decrease in viral titers *in vivo* (Figure 3(b)) and *in vitro* (Figure 1). These results indicate that Ekybion possesses both antiviral and immunomodulatory properties during influenza A infection.

**Figure 5:**
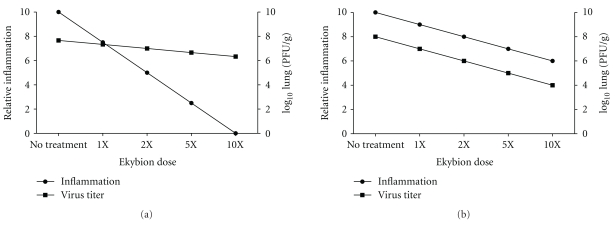
Theoretical mechanism of action and method for improved efficacy. (a) The current composition of Ekybion contains plant extracts known to have antiviral and immunomodulatory potential. The beneficial and negative interplay between these two effects is shown here where mild inhibition of inflammation (resulting in relief of some symptoms associated with influenza infection) is beneficial but overt inhibition of inflammation (impaired tissue repair or viral clearance late in infection) is detrimentla to disease resolution. Conversely, increasing inhibition of virus replication is beneficial. (b) In order to maximize the therapeutic benefit obtained from Ekybion we propose optimizing the concentrations of the 16 components to increase the antiviral properties and limit the immunomodulatory effects.

 Based on our observations, we propose the following mechanism by which Ekybion improves survivorship (Figure 5(a)). For the 1X treatment group, although we observed no reduction in virus titer *in vivo* at this dose, we did observe a mild anti-inflammatory effect which may have provided some relief of symptoms associated with an influenza infection. This observation is similar to the results obtained for a natural compound, Glycyrrhizin, in the treatment of influenza infection. Glycyrrhizin, which is present in liquorices roots and used in a number of traditional cough remedies, reduces the severity of infection in mice infected with influenza A H2N2 and reduced the lethality of the infection but did not affect virus replication [43]. In the case of higher doses of Ekybion (10X for 2 min or 1X for 4 min), there was a beneficial decrease in virus load, but any benefit was overshadowed by excessively impaired inflammation (Figure 5(a)), especially with respect to the important antiviral cytokine IFN-*γ* which has been shown to affect survival of mice infected with influenza A, but without affecting viral titers [44]. Instead, IFN-*γ* has been shown to play a critical role in shaping the production and infiltration of NK cells early in infection and T-cell infiltration later in influenza infection, thus reducing the amount of tissue damage while still leading to proper viral clearance [44]. We therefore hypothesize that at higher doses of Ekybion, mice died due to an unbalanced immune response which resulted in either increased tissue damage or impaired viral clearance later in infection than examined in this paper. Furthermore, the 1X 4 min treatment group exhibited additional signs of stress from extended restraint during treatment thus adding to the mortality seen in this group. These findings bring into stark relief the delicate balancing act of modulating immune responses during an infection. Future research with Ekybion will greatly benefit from examining the antiviral and anti-inflammatory effects of the 16 compounds separately and then determining the concentration of each compound which will provide maximum benefit for virus inhibition and modulation of inflammation (Figure 5(b)).

## Figures and Tables

**Table 1:** Composition of Ekybion.

Plant extracts	*Arnica montana, Baptisia tinctoria*,* Bryonia alba*,* Lytta vesicatoria*,* Echinacea angustifolia, Phellandrium aquaticum*,* Pulsatilla nigricans*,* Sepia officinalis*, and *Thuja occidentalis *

Inorganic compounds	nitric Acid, phosphorus, calcium phosphate, potassium bisulphate, calcium hydrate, calcium sulfide, iodine, and silicic acid

**Table 2:** Survivorship for Balb/c mice treated with Ekybion.

Treatment start	Administration time (Ekybion conc.)	Survivorship live/total	Mean day of death ± SD	Log rank test
3 hour pre	2 min (control)	0/13	9.23 ± 2.13	
3 hour pre	2 min (1X)	6/13	9.86 ± 1.77	*P* = .0078
3 hour pre	4 min (1X)	0/6	10.0 ± 1.67	
3 hour pre	2 min (10X)	1/6	9.20 ± 1.10	
6 hour post	2 min (1X)	0/7	9.43 ± 2.37	

Eight-week-old female Balb/c mice were infected with 5 MLD_50_ of influenza A/PR/8/34 H1N1 and treated three times daily for 10 days.
